# Successful aging: insights from proteome analyses of healthy centenarians

**DOI:** 10.18632/aging.102826

**Published:** 2020-02-25

**Authors:** Alejandro Santos-Lozano, Pedro L. Valenzuela, Francisco Llavero, Simone Lista, Pedro Carrera-Bastos, Harald Hampel, Helios Pareja-Galeano, Beatriz G. Gálvez, Juan Antonio López, Jesús Vázquez, Enzo Emanuele, José L. Zugaza, Alejandro Lucia

**Affiliations:** 1 Research Institute of the Hospital 12 de Octubre ("imas12"), Madrid, Spain; 2 i+HeALTH, European University Miguel de Cervantes, Valladolid, Spain; 3 Systems Biology Department, University of Alcalá, Madrid, Spain; 4 Achucarro Basque Center for Neuroscience, Science Park of the UPV/EHU, Leioa, Spain; 5 Sorbonne University, GRC no. 21, Alzheimer Precision Medicine (APM), Pitié-Salpêtrière Hospital, Paris, France; 6 Brain and Spine Institute (ICM), Paris, France; 7 Institute of Memory and Alzheimer's Disease, Department of Neurology, Pitié-Salpêtrière Hospital, Paris, France; 8 Centre for Primary Health Care Research, Lund University/Region Skane, Skane University Hospital, Malmo, Sweden; 9 Nutriscience – Education and Consulting, Lisbon, Portugal; 10 Faculty of Sport Sciences, European University of Madrid, Madrid, Spain; 11 Laboratory of Cardiovascular Proteomics, Centro Nacional de Investigaciones Cardiovasculares Carlos III (CNIC), Madrid, Spain; 12 Centro Integrado de Investigación Biomédica en Red en Enfermedades Cardiovasculares (CIBERCV), Madrid, Spain; 13 2E Science, Robbio, Italy; 14 Department of Genetics, Physical Anthropology and Animal Physiology, Faculty of Science and Technology, UPV/EHU, Leioa, Spain; 15 IKERBASQUE, Basque Foundation for Science, Bilbao, Spain; 16 Centro de Investigación Biomédica en Red, Fragilidad y Envejecimiento Saludable (CIBERFES), Madrid, Spain

**Keywords:** proteomics, healthy aging, elderly, senescence, immune system

## Abstract

Healthy aging depends on a complex gene-environment network that is ultimately reflected in the expression of different proteins. We aimed to perform a comparative analysis of the plasma proteome of healthy centenarians (n=9, 5 women, age range 100–103 years) with a notably preserved ambulatory capacity (as a paradigm of ‘successful’ aging), and control individuals who died from a major age-related disease before the expected life expectancy (n=9, 5 women, age range: 67–81 years), and while having impaired ambulatory capacity (as a paradigm of ‘unsuccessful’ aging). We found that the expression of 49 proteins and 86 pathways differed between the two groups. Overall, healthy centenarians presented with distinct expression of proteins/pathways that reflect a healthy immune function, including a lower pro-inflammatory status (less ‘inflammaging’ and autoimmunity) and a preserved humoral immune response (increased B cell-mediated immune response). Compared with controls, healthy centenarians also presented with a higher expression of proteins involved in angiogenesis and related to enhanced intercellular junctions, as well as a lower expression of proteins involved in cardiovascular abnormalities. The identification of these proteins/pathways might provide new insights into the biological mechanisms underlying the paradigm of healthy aging.

## INTRODUCTION

The aging process is characterized by progressive changes to the integrity of body tissues and systems [1]. Notably, aging is associated with chronic systemic inflammation (CSI, as reported in various cohorts from industrialized countries) [2]. CSI is not the only immune response altered by aging, since both innate and adaptive immune responses are affected [3]. The cardiovascular system is another important target of the aging process [4], which combined with CSI leads to a higher risk of cardiovascular disease (CVD) [2], representing the main cause of death in people older than 65 years [5]. Finally, another feature of aging is a change in cell barrier integrity, which is essential to maintain cellular homeostasis [6, 7].

Centenarians are the survival tail of the population and represent – especially if free of major diseases – a good model of healthy (or 'successful') aging since they have escaped diseases of the pre-antibiotic era, and have evaded (or at least postponed) age-related diseases and their fatal consequences. In this respect, blood proteome analysis might provide important information on causal mechanisms of healthy aging because it reflects both inherent (genetics) and environmental factors [8], and can help to reveal the protein networks and individual protein candidates involved in disease or aging processes [9]. However, to our knowledge no previous study has assessed the plasma proteome of healthy centenarians.

It was the purpose of this study to perform a novel, comparative analysis of the plasma proteome of healthy centenarians – as a paradigm of successful aging – and controls from the same geographic location who died from a major age-related disease before the expected life expectancy – as a paradigm of unsuccessful aging. We hypothesized that a more favorable ‘aging proteome profile’ (e.g., at the inflammatory and cardiovascular level) together with differential expression of some specific candidate proteins, would be identified in centenarians compared with controls.

## RESULTS

The participants’ main characteristics are shown in Table 1. Healthy centenarians were significantly older (by 25 years on average) than controls but had a more preserved ambulatory capacity (p<0.001).

**Table 1:** Clinical characteristics of healthy centenarians and controls.

**Group**	**Sex**	**Status**	**Cause of death**	Age (years)*	**Clinical characteristics**	FAC*
Healthy centenarians	Male	Alive	-	101	Hearing impairment	3
	Male	Alive	-	100	Visual impairment	3
	Male	Alive	-	102	Nothing significant	2
	Male	Alive	-	102	Hearing impairment	3
	Female	Alive	-	101	Nothing significant	2
	Female	Alive	-	103	Visual and hearing impairment	3
	Female	Alive	-	101	Visual impairment	3
	Female	Alive	-	100	Nothing significant	3
	Female	Alive	-	101	Visual and hearing impairment	3
				Mean (SD): 101 (1)		Median (IQ): 3 (0.5)
						
Controls	Male	Died	Stroke	67	Stroke, cognitive disturbances	2
	Male	Died	Aortic aneurysm rupture	79	Peripheral artery disease, transient ischemic attacks	2
	Male	Died	Multi-organ failure	75	Frailty, cognitive decay, sarcopenia	0
	Male	Died	Acute myocardial infarction	81	Cognitive decay, acute myocardial infarction	2
	Female	Died	Stroke	73	Femur fracture, syncope, stroke	1
	Female	Died	Stroke	72	Behavioral disturbances, memory loss, stroke	1
	Female	Died	Traumas after fall	74	Vertebral fractures, osteoporosis, fall	0
	Female	Died	Sudden cardiac death	68	Behavioral disturbances, transient ischemic attacks	2
	Female	Died	Acute myocardial infarction	81	Frailty, acute myocardial infarction	2
				Mean (SD): 74 (5)		Median (IQ): 2 (1.5)

Abbreviations: FAC, Functional ambulatory capacity (see text for explanations); IQ, interquartile range; SD, standard deviation. Symbol: * *p*≤0.001 for the comparison between the two groups.

A total of 949 human proteins were identified by liquid chromatography coupled to tandem mass spectrometry, of which 798 valid protein entries were used in the next steps of the study and 151 were filtered out (24 corresponded to keratin, 17 proteins could not be traced to a valid reviewed human UniProt, and 110 were traced to the same reviewed human UniProt entry as other more reliable identified protein).

### Protein candidates

Forty-nine plasma proteins showed significantly differential expression between the two study groups (false discovery rate [FDR] q-value <0.05), of which 17 and 32 were upregulated and downregulated, respectively, in healthy centenarians compared with controls (Supplementary Table 1). The 10 proteins that showed a greatest degree of differential expression (i.e., Log2 fold change (FC) >1.95 and false discovery rate [FDR] q-value <0.05) between centenarians and controls and were thus considered to be the best candidates were: C-type lectin domain family 3 member B (CLEC3B), cysteine-rich secretory protein 3 (CRISP3), insulin like growth factor binding protein (IGFALS), taste 1 receptor member 3 (TAS1R3), and transforming growth factor beta induced (TGFBI) – which were upregulated in healthy centenarians compared to their unhealthy controls, and thus potentially associated with healthy (successful) aging; and aminopeptidase (AOPEP), complement component 1S (C1S), cluster of differentiation 14 (CD14), cyclin dependent kinase like 1 (CDKL1), and cartilage acidic protein 1 (CRTAC1) – which were downregulated in the aforementioned group and thus potentially associated with the opposite phenotype, unhealthy or unsuccessful aging.

On the other hand, when analyzing male and female data separately, no differentially expressed proteins were found between healthy centenarians and controls using the same statistical threshold, likely due to the smaller number of samples analyzed.

### Gene set enrichment analysis

**Figure 1:**
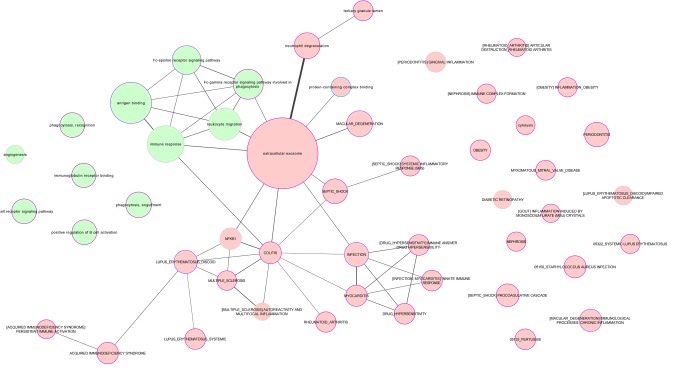
**Representation of the biological relationships between healthy and unhealthy aging by subnetworks.** The processes shared by 50 or more common proteins (i.e., those reflecting the strongest relationships) are shown. Nodes are filled in green and red background for proteins upregulated and downregulated, respectively, in healthy, functional centenarians compared with a control group with the opposite phenotype (i.e., mostly composed of people with low functional independence and who died from cardiovascular disease before becoming an octogenarian).

The level of expression of the 798 proteins was successfully transformed into the signal-to-noise ratio ranking parameter (according to gene set enrichment analysis [GSEA] software) and was used in the GSEA enrichment analyses. A total of 86 enriched pathways (FDR q-values <0.1) were found, of which 22 and 64 were upregulated and downregulated, respectively, in healthy centenarians compared with controls (Table 2, see also Figure 1 for a network summary).

**Table 2:** Number of enriched pathways identified in the global and sex-specific analysis according to the false discovery rate (FDR) threshold value applied.

	**Global**		**Men**		**Women**	
**Cohort**	**FDR < 0.1**	**FDR < 0.05**	**FDR < 0.1**	**FDR < 0.05**	**FDR < 0.1**	**FDR < 0.05**
Healthy centenarians	11	8	8	4	19	12
Controls	38	28	5	4	58	37

To gain insight into the molecular mechanisms associated with healthy or successful aging, proteins were grouped into the following biological processes: *(i)* immune system-related pathways (Supplementary Table 2); *(ii)* cell junctions (Table 3); *(iii)* cardiovascular system abnormalities (angiogenesis, coagulation and CVD; Table 4); and *(iv)* other processes (Table 5: intracellular mechanisms [i.e., ATPase and microtubule motor activity, MAPK signaling pathway], metabolic alterations, and age-related diseases, as well as other conditions). Overall, centenarians presented with enrichment in general immune response mechanisms, B cell-mediated immune responses and phagocytosis whereas the opposite trend was found for T cell-mediated immune response, complement-mediated cytolysis, and immune system-related pathological states such as CSI (also known as ‘inflammaging’ when it affects old people), autoimmunity, infection and other immune response-related diseases. The expression of proteins involved in cell junction integrity was also upregulated in healthy centenarians compared with controls, and the former showed a higher expression of proteins involved in angiogenesis and a lower expression of proteins involved in coagulation and CVD.

**Table 3:** Cell junction-related processes differentially expressed in healthy centenarians compared with controls.

**Dataset source**	**Process Name**	***Global FDR***	***Men FDR***	***Women FDR***	**Related process**	**Expression compared with controls**
BEDPATH	Squamous epithelial oral cell keratinized tight junction	-	**0.042**	**0.040**	Cell junctions	↑
BEDPATH	Pneumocyte type II tight junction	-	**0.047**	**0.035**	Cell junctions	↑
BEDPATH	Endothelial tight junctions	-	**0.048**	0.063	Cell junctions	↑
BEDPATH	Lung basal cell tight junction	-	0.069	**0.035**	Cell junctions	↑
BEDPATH	Squamous epithelial ectocervix basal cell tight junction	-	-	**0.042**	Cell junctions	↑
BEDPATH	Tight junctions in non-keratinized epithelium	-	0.052	0.050	Cell junctions	↑

FDR q-values <0.5 are highlighted in bold, and those between 0.05 and 0.1 are in grey font. Symbol: ↑, higher expression in healthy centenarians compared with controls (also denoted by green).

**Table 4:** Cardiovascular system-related processes differentially expressed in healthy centenarians compared with controls.

**Data source**	**Process Name**	***Global FDR***	***Men FDR***	***Women FDR***	**Related Process**	**Expression compared with controls**
GOPROCESS	Angiogenesis	**-**	-	0.058	Angiogenesis	↑
BEDMOTIVE	[Septic_Shock] Procoagulative Cascade	**0.006**	-	**<0.001**	Coagulation	↓
GOLOCATION	Extracellular exosome	0.068	-	**0.023**	Coagulation	↓
KEGG	04610_Complement and coagulation cascades	-	-	0.059	Coagulation	↓
BED	Thrombophlebitis	-	-	0.059	Coagulation	↓
BED	Myxomatous mitral valve disease	**0.008**	-	**0.005**	CVD	↓
BED	Myocardial infarction	-	-	**0.030**	CVD	↓
BED	Stroke	-	-	**0.044**	CVD	↓

FDR q-values <0.5 are highlighted in bold, and those between 0.05 and 0.1 are in grey font. Symbols: ↑, ↓ higher and lower expression in healthy centenarians, respectively, compared with controls (also denoted by green and red, respectively).

**Table 5:** Other processes differentially expressed between healthy centenarians and controls.

**Dataset source**	**Process name**	***Global FDR***	***Men FDR***	***Women FDR***	**Related process**	**Expression compared with controls**
GOFUNCTIONID	ATPase activity	-	-	0.062	Intracellular mechanisms	↑
GOFUNCTIONID	Microtubule motor activity	-	-	0.071	Intracellular mechanisms	↑
KEGG	04010_MAPK signaling pathway	-	-	0.070	Intracellular mechanisms	↓
KEGG	Response to ethanol	-	-	0.071	Metabolic alterations	↓
BED	Osteoarthritis	-	-	0.095	Age-related and other diseases	↓
BED	Non-alcoholic fatty liver disease	-	-	0.053	Age-related and other diseases	↓
BED	Acne vulgaris	-	-	0.080	Age-related and other diseases	↓

FDR q-values <0.5 are highlighted in bold, and those between 0.05 and 0.1 are in grey font. Symbols: ↑, ↓ higher and lower expression in healthy centenarians, respectively, compared with controls (also denoted by green and red, respectively).

## DISCUSSION

The likelihood of healthy aging is dependent upon complex gene-environment interactions that are ultimately reflected in the control of expression of different proteins. In this respect, the present study identifies a number of proteins whose expression levels in plasma seem to differ between healthy centenarians with largely preserved ambulatory capacity − who can be considered a paradigm of healthy or successful aging − and a control group of individuals who had an unsuccessful aging process (*i.e.*, died before the expected mean life expectancy, before reaching the status of ‘oldest old’ [≥85 years] due to an age-related disease, mainly CVD, and while having an impaired ambulatory capacity). The 10 ‘best’ candidate proteins, that is, those showing the greatest difference in expression levels between healthy centenarians and controls and which could therefore be of special interest for future replication studies at the individual protein level were CLEC3B, CRISP3, IGFALS, TAS1R3, and TGFBI (all been potentially associated with successful aging) and AOPEP, CD14, CDKL1, and CRTAC1 (unsuccessful aging). CELC3B (also known as tetranectin) is s thought to participate in plasminogen activator–related biological processes such as tissue degradation/ remodeling and extracellular proteolysis and a missense variant in CELC3B gene (p.S106G) has been recently associated with extreme longevity (reaching 95+ years) in East Asian populations [10]. To the best of our knowledge, no direct/indirect evidence with regard to extreme longevity or successful aging has been reported for the rest of these candidate proteins.

Previous research has attempted to identify the proteomic signatures of aging [11, 12] (see Supplementary Table 1 for a comparison with our main results). Some proteins that were significantly downregulated in healthy centenarians and thus theoretically associated with unsuccessful aging according to our results – albumin, beta 2 microglobulin (B2M), complement component 9 (C9), cystatin 3 (CST3), EGF-containing fibulin-like extracellular matrix protein 1 (EEMP1), lysozyme, and serpin peptidase inhibitor, clade G (C1 inhibitor), member 1 (SERPING1) − were recently shown to be positively correlated with *chronological* age in a cohort of healthy men and women aged 22–93 years [12]. In the aforementioned report, TGBI – which in our study was a potential biomarker of successful aging – was also correlated with *chronological* age Thus, although differences in study design make comparisons difficult, taken together the present findings and those of previous research with old – albeit non-centenarian – adults [11, 12] suggest that *(i)* the expression levels of these proteins (albumin, B2M, C9, CST3, EEMP1, lysozyme, SERPING1, TGFBI) usually increase with age in the overall population, and yet *(ii)* an attenuation of this increasing trend – although maybe not for TGFBI – might be a signature of reaching extremely advanced ages in good health conditions. In turn, our findings that the expression levels of serpin family F member 2 (SERPINF2, also known as alpha 2- antiplasmin) were significantly elevated in healthy centenarians compared to old but non-centenarians controls add complementary information to the inverse correlation with chronological age (β=-0.026) found for this protein in the study by Tanaka et al [12], and might suggest that this protein is associated with chronological – but not necessarily ‘unhealthy’ – aging. SERPINF2 acts as a major inhibitor of plasmin, which degrades fibrin and other proteins involved in blood clotting. Higher levels of α2-antiplasmin have been reported in healthy people compared to age-matched individuals with myocardial infarction [13], which could support the present findings of higher SERPINF2 levels in healthy centenarians compared to diseased controls. However, other authors found a positive relationship between α2-antiplasmin levels and the risk of myocardial infarction or coronary heart disease [14, 15], although in one of these studies the relationship was due to a positive association between α2-antiplasmin levels and other CVD risk factors (i.e., blood pressure, cholesterol levels) [15]. Thus, further research in larger and more varied cohorts and including replication analyses is needed to clarify the influence of SERPINF2 on both aging and cardiovascular health.

The observed proteomic changes overall reflect that healthy centenarians present with a higher expression of proteins involved in angiogenesis and cell junction integrity, but with a reduction in those involved in the incidence of CVD. Regarding cell junctions, we found that healthy centenarians showed a higher expression of proteins related to enhanced paracellular barrier function. This is relevant because intercellular interactions play a major role in establishing the architecture of tissues and organs, and consequently in the regulation of cell proliferation and migration, as well as in maintaining tissue barrier homeostasis and function in different organs [6]. Indeed, disruption of cell junctions is associated with numerous conditions such as neuronal dysfunction and neurodegeneration [16], sarcopenia (muscle atrophy) [17], cardiomyopathies [18], CSI [19], type 2 diabetes [19], CVD [20], and even autoimmunity [21].

With respect to immune system-related processes, previous studies have shown that both adaptive and innate immune responses become deregulated with aging in a process known as ‘immunosenescence’ [3], which is partially characterized by changes in the cytokine *milieu* that can lead to ‘inflammaging’ [22]. Although inflammation per se is an evolutionary conserved survival phenomenon, when it becomes chronic and systemic (i.e., CSI) it might contribute to many of the deleterious processes that occur during the aging process (e.g. sarcopenia, osteoporosis, type 2 diabetes, and vascular and neurological conditions) [2, 23]. In this regard, we found higher expression levels of proteins involved in the ‘inflammaging’ process and of those involved in complement-mediated cytolysis, infection and autoimmunity in the control group, whereas healthy centenarians had a higher expression of proteins related to B lymphocyte-mediated immune response and phagocytosis. These findings fit well with previous studies supporting the role of a healthy immune system as a differential characteristic of disease-free centenarians [23–25].

Another major finding of our study was that healthy centenarians presented higher expression levels of proteins involved in angiogenic processes, as well as of those involved in a lower incidence of CVD and coagulation. It has been suggested that the increased incidence of CVD with aging might be partly due to a decreased capillary density as well as to an impaired angiogenic response to hypoxic stimuli (*i.e.*, reduced availability of endothelial nitric oxide, and decreased hypoxia-inducible factor 1-alpha activation and expression of angiogenic factors) [26], which would support the observed role of an enhanced angiogenic capacity as a distinguishing feature of healthy centenarians.

There are some limitations to our study. Contrary to previous research ([see Landen et al. [27] for a review), we found no sex-specific differences in plasma proteins between centenarians and controls, which could be partly due to the low sample size analyzed. Our results – particularly at the individual protein level – should be replicated in larger cohorts using different methodologies (e.g., enzyme-linked immunosorbent assay) and including individuals with different ages and health status. Moreover, although we highlight different proteins whose expression levels differed between healthy centenarians and controls, we cannot distinguish whether this was due to a genetic or an environmental influence. Indeed, some genetic factors are associated with exceptional longevity [28–30]. However, we consider that this might be, in turn, a strength of our study when compared with the analysis of other factors (e.g., genetics, lifestyle) alone, as it enables the association of both inherent and external factors together (reflected in the expression of a given protein) with exceptional, healthy longevity. It must also be noted that proteins were analyzed using whole-plasma samples, but whether their expression varies depending on specific cell types should be elucidated. In turn, a major strength of our study is the choice of a unique group of healthy, largely functional centenarians as a paradigm of healthy aging and the comparison with individuals showing virtually the opposite phenotype. On the other hand, our study has some potential clinical implications. First, the proteome of healthy centenarians remains largely unknown up to date. Thus, the present findings might provide preliminary data that could be used in future research aimed to predict at the proteome level the likelihood of reaching extreme longevity in good health conditions. In addition, since a ‘proteomic hallmark’ of healthy centenarians was mainly attenuation of inflammaging and improved immune function, more efforts are needed in the promotion and implementation of lifestyle interventions that can reduce CSI and improve immune function in old people, such as regular physical exercise [31], tobacco smoking cessation [32], sleep improvement [33], body fat loss [34], healthy dietary patterns (diets rich in vegetables and fruits [35] or the Mediterranean diet, which is rich in vegetables and fruits but also in nuts, legumes, fish, and ‘healthy’ dietary fats) [36], and certain nutritional compounds, including mainly probiotics and omega-3 [37].

In summary, we have identified a number of potential protein biomarkers and related molecular processes and pathways that differ between healthy centenarians and a gender-matched control group of old individuals who died before reaching the status of oldest old. Our results seem to confirm our hypothesis that centenarians have a healthier immune system function characterized by an attenuation of the “inflammaging” phenotype and a preserved humoral immune response. Moreover, they present with higher levels of proteins involved in angiogenesis and in the reduced incidence of cardiovascular abnormalities, as well as of those related to an enhanced intercellular junction function. The proteins/pathways found here for healthy centenarians might provide new insights into the understanding of the unique biological characteristics that characterize these individuals, which might help to identify the 'proteomic profile’ associated with successful aging.

## MATERIALS AND METHODS

### Participants

All participants or their legal representatives provided written informed consent. The study was conducted in accordance with the Declaration of Helsinki and was approved by the local Institutional Review Board (reference #16/081). At this discovery stage, the sample size was determined expecting a signal of 5 standard deviations (SDs) and an statistical power (1- β) of 0.80 with at least 80% of expression in the healthy centenarians group [39].

We studied healthy centenarians (n=9 [5 female]; age range: 100–103 years) from the North of Italy (Lombardy) who were free of any clinically relevant condition other than the expected visual or hearing impairment (Table 1). Further, all of them showed a notably preserved ambulatory ability, with the majority (n=7, ~78%) showing a score of 3 in the 0 to 4 Functional Ambulation Classification (FAC) scale [38], that is, they were able to walk independently with verbal supervision/guarding and without manual contact. Only two of them required light or intermittent manual contact during walking (FAC score of 2).

Participants in the control group recruited from the same area (n=9 [5 female]; age range: 67 to 81 years) showed the opposite phenotype; that is, they all died before the mean expected life expectancy for Italy (~83 years), and the great majority (n=8, ~89%) died of a CVD before (n=6) or shortly after (n=2) becoming an octogenarian. Further, in contrast to healthy centenarians, they showed a remarkable functional impairment, with two subjects actually unable to walk (FAC score of 0) and two needing firm, continuous manual support from one person to assist balance (FAC score of 1). Blood sampling was performed on average 32 ± 19 weeks before the day of death.

### Proteomic analysis

### Plasma sample preparation

Fasting venous blood samples were collected from the antecubital vein in EDTA- treated vials in the morning after an overnight fast (08:00 to 09:00 am) and were kept at 4°C until preparation to prevent coagulation and minimize protein degradation. The specimens were then centrifuged at 1500 g for 10 minutes at 4°C. Supernatants were transferred to new tubes as aliquots. Ten microliters of protease inhibitor were added to each 1.0 mL plasma aliquot, which were then stored at -80°C for later analysis and only one freeze-thaw cycle was allowed. All samples were prepared within 1 hour of sample collection and showed no signs of hemolysis.

### Protein digestion and isobaric labeling

For the quantitative differential analysis by liquid chromatography coupled to tandem mass spectrometry (LC-MS/MS) using isobaric tags (TMT 10-plex), 100 μg of total protein was digested using the filter-aided protocol as previously described [40] with minor modifications. Proteins were reduced in 7 M urea and 0.1 mM Tris–HCl (pH 8.5) (UA) containing 5 mM TCEP (tris[2-carboxyethyl]phosphine), and loaded onto 10 kDa centrifugal filter devices (NanoSep 10k Omega, Pall Life Sciences; Port Washington, NY). The buffer was replaced by washing the filters with UA, and proteins were then alkylated using 20 mM iodoacetamide in UA for 20 minutes in the dark. The excess alkylating reagent was eliminated by washing three times with UA and three additional times with 50 mM ammonium bicarbonate. Proteins were digested overnight at 37°C with modified trypsin (Promega Biotech Ibérica; Alcobendas, Madrid, Spain) in 50 mM ammonium bicarbonate at 30:1 protein:trypsin (w/w) ratio. The resulting peptides were eluted by centrifugation with 50 mM ammonium bicarbonate (twice) and 0.5 M sodium chloride. Trifluoroacetic acid was added to a final concentration of 1% and the peptides were desalted onto C18 Oasis-HLB cartridges (Waters; Milford, MA) and dried-down for further analysis.

For stable isobaric labeling, the tryptic peptides were dissolved in 150 mM tri-ethyl-ammonium bicarbonate buffer, and the peptide concentration was determined by measuring amide bonds with the Direct Detect system (Millipore, Ibérica). Equal amounts of each peptide sample were labeled using 10-plex TMT Reagents (Thermo Fisher Scientific; Waltham, MA), which were previously reconstituted with 70 μl of acetonitrile. After incubation at room temperature for 1 h, the reaction was stopped with 0.5% trifluoroacetic acid, incubated for 10 minutes, and peptides were combined. Samples were concentrated in a Speed Vac, desalted onto C18 Oasis-HLB cartridges and dried-down for further analysis. To increase proteome coverage, TMT-labeled samples were fractionated by high-pH reverse phase chromatography (High pH Reversed-Phase Peptide Fractionation Kit, Pierce; Rockford, IL) and concentrated as before.

### Protein identification and quantification

Labeled peptides were analyzed by LC-MS/MS using a C-18 reversed phase nano-column (75 μm I.D. × 50 cm, 2 μm particle size, Acclaim PepMap RSLC 100 C18; Thermo Fisher Scientific) in a continuous acetonitrile gradient consisting of 0–30% B for 240 minutes and 50–90% B for 3 minutes (A = 0.1% formic acid; B = 95% acetonitrile, 0.1% formic acid). A flow rate of 200 nL min^-1^ was used to elute peptides from the nano-column to an emitter nanospray needle for real time ionization and peptide fragmentation on an Orbitrap Fusion mass spectrometer (Thermo Fisher Scientific). An enhanced FT-resolution spectrum (resolution = 70,000 at m/z 200) followed by the MS/MS spectra from the Nth most intense parent ions were analyzed along the chromatographic run. Dynamic exclusion was set at 40 seconds.

For peptide identification, all spectra were analyzed with Proteome Discoverer (version 2.1.0.81) using SEQUEST-HT (both from Thermo Fisher Scientific). For database searching at the Uniprot database containing all sequences from human and contaminants (8^th^ November 2016; 70,902 entries), the following parameters were selected: trypsin digestion with two maximum missed cleavage sites, precursor and fragment mass tolerances of 2 Da and 0.02 Da, respectively, carbamidomethyl cysteine, and TMT modifications at N-terminal and Lys residues as fixed modifications, and methionine oxidation as dynamic modification. Peptide identification was performed using the probability ratio method [41] and false discovery rate (FDR) was calculated using inverted databases and the refined method [42] with additional filtering for precursor mass tolerance of 15 ppm [43].

Identified peptides with an FDR equal to or lower than 1% were used to quantify the relative abundance of each protein from reporter ion intensities, and statistical analysis of quantitative data was performed using the WSPP statistical model previously described [44]. In this model, protein log2-ratios are expressed as standardized variables; that is, in units of standard deviation (SD) according to their estimated variances (Zq values).

### Functional protein analysis

Functional protein analysis of the whole set of quantified proteins was performed using a novel algorithm system biology triangle, developed specifically for the analysis of coordinated protein responses in high-throughput quantitative proteomics experiments [45]. This algorithm correlates the performance of a group of proteins inside a category (biological process) in terms of their quantitative behavior (relative abundance); thus, changes can be detected in functional biological processes far beyond individual protein responses. Variations in the abundance of annotated functional categories were visualized by comparing the cumulative frequency (sigmoid) plots of the standardized variable with that of the normal distribution, as in previous research [46]. Individual protein changes were also considered for further analysis.

### Statistical analysis

### Protein candidates

Data normality was analyzed using the Kolmogorov-Smirnov test. Age and FAC values in the two groups were compared with the Mann Whitney *U* test. To identify proteins in plasma profile differentially expressed between healthy centenarians and controls, independent samples Student’s *t-*tests or Wilcoxon-rank sum tests were applied depending on data normality. Multi-test correction was performed according to the Benjamini-Hochberg method, that is, *p*-values were adjusted with FDR correction [47]. The analysis was repeated for the whole proteomic data and independently from men’s and women’s samples. Statistical analyses were performed with SPSS 22.0 (IBM, Chicago, IL).

### Proteomics-based analysis of protein networks

### Processing of protein expression data

The proteins identified in the plasma profiles were filtered to unique human reviewed protein entries according to UniProt KB [48]. To reduce a potential interference for high signals, the keratin protein entries were excluded from the analysis. Also, the protein entries that were traced to an unreviewed UniProt entry were manually curated to find a valid reviewed one.

When more than one protein entry was traced to the same reviewed UniProt entry, the following criteria were applied to prevent duplications and to obtain a list of unique proteins: *(i)* to prioritize protein entries with an identical protein ID to the one noted in UniProt KB; *(ii)* to prioritize protein entries that were automatically traced to the reviewed UniProt entry over those that were first traced to an unreviewed or deleted one and then manually curated; *(iii)* to prioritize protein entries with valid signals for all the male and female samples; *(iv)* to prioritize whole protein entries over those referring to fragments; and *(v)* to apply an alphabetical criterion when all the criteria above did not apply.

### Gene set enrichment analysis

Proteomic data were analyzed using the Gene Set Enrichment Analysis (GSEA) tool [49] to compare the differential pathways and molecular processes between the plasma samples of healthy centenarians and controls. This approach categorizes the genes according to a specific metric, ranking the most statistically significant genes at the top end of the list. The signal-to-noise ratio for each protein was used for the ranking parameter analysis. A significance threshold was defined at FDR q-value <0.1.

Specifically, as performed elsewhere [9], the enrichment was run over the following databases: Gene Ontology (GO) terms (Biological Process, Cellular Component, Molecular Function), Kyoto Encyclopedia of Genes and Genomes (KEGG) pathways, pathological conditions and motives included in the Biological Effector Database (BED) (Anaxomics proprietary database), PharmGKB pathways [50], the pathways from Small Molecule Pathway Database (SMPDB), and the regulatory molecular mechanisms included in Transcriptional Regulatory Relationships Unraveled by Sentence-based Text-mining (TRRUST) database. Entries with no signal in samples for the particular analysis (all samples in global analysis and sex-exclusive for male-exclusive and female-exclusive analyses) were excluded from the GSEA.

### Visualization as a network of the relationship between enriched pathways

Cytoscape 3.5.1. software was used to represent the relationship between the enriched pathways identified by GSEA (based on the number of common annotated genes between datasets).

## Supplementary Material

Supplementary Table 1

Supplementary Table 2
