# Supplementary material for: Successful aging: insights from proteome analyses of healthy centenarians
Source: Aging (Albany NY). 2020 Feb 25;12(4):3502–15. doi: 10.18632/aging.102826 (PMC7066932; doi:10.18632/aging.102826)
Supplement: Supplementary Table 1 [file aging-12-102826-s001..docx]

**Supplementary Table 1.** List of proteins differentially expressed in healthy centenarians compared with the control group.

| **UniProt ID** | **Gene Name** | **Normality** | **FDR t-Test** | **FDR Wilcoxon** | **Log2 FC** |
| --- | --- | --- | --- | --- | --- |
| P04217 | A1BG | ✓ | **0.009** | 0.022 | -1.87 |
| Q8NE71 | ABCF1 | ✓ | **0.022** | 0.026 | 1.68 |
| Q8N9L9 | ACOT4 | 🗶 | 0.013 | **0.026** | 1.69 |
| P43652 | AFM | 🗶 | 0.11 | **0.032** | 2 |
| P02768 | ALB | ✓ | **0.022** | 0.022 | 1.79 |
| P02760 | AMBP | ✓ | **0.006** | 0.01 | -1.75 |
| Q8N6M6 | AOPEP | ✓ | **0.012** | 0.014 | -1.97 |
| P32121 | ARRB2 | ✓ | **0.031** | 0.042 | -1.5 |
| P25311 | AZGP1 | ✓ | **0.013** | 0.014 | -1.71 |
| P61769 | B2M | ✓ | **0.009** | 0.007 | -1.59 |
| P09871 | C1S | ✓ | **0.047** | 0.047 | -2.18 |
| P01031 | C5 | ✓ | **0.009** | 0.02 | -1.88 |
| P10643 | C7 | ✓ | **0.009** | 0.014 | -1.87 |
| P02748 | C9 | 🗶 | 0.009 | **0.022** | -1.6 |
| P08571 | CD14 | ✓ | **0.01** | 0.022 | -2.1 |
| Q00532 | CDKL1 | ✓ | **0.045** | 0.07 | -1.97 |
| P05452 | CLEC3B | ✓ | **0.039** | 0.047 | 2.06 |
| Q8N8Q8 | COX18 | ✓ | **0.009** | 0.014 | 1.66 |
| P54108 | CRISP3 | ✓ | **0.022** | 0.022 | 2.29 |
| Q9NQ79 | CRTAC1 | ✓ | **0.006** | 0.01 | -1.96 |
| P01034 | CST3 | ✓ | **0.006** | 0.007 | -1.65 |
| Q16610 | ECM1 | ✓ | **0.03** | 0.042 | 1.93 |
| Q12805 | EFEMP1 | ✓ | **0.01** | 0.026 | -1.56 |
| P02790 | HPX | ✓ | **0.031** | 0.047 | -1.8 |
| P35858 | IGFALS | ✓ | **0.039** | 0.056 | 2.1 |
| P01593 | IGKV1D-33 | ✓ | **0.039** | 0.07 | 1.66 |
| Q9H160 | ING2 | 🗶 | 0.01 | **0.022** | -1.63 |
| P20701 | ITGAL | ✓ | **0.01** | 0.014 | -1.44 |
| Q06033 | ITIH3 | ✓ | **0.001** | 0.007 | -1.57 |
| P13796 | LCP1 | 🗶 | 0.022 | **0.026** | -1.5 |
| P02750 | LRG1 | ✓ | **0.028** | 0.047 | -1.43 |
| P61626 | LYZ | ✓ | **0.009** | 0.014 | -1.81 |
| Q9UKX3 | MYH13 | ✓ | **0.01** | 0.014 | 1.76 |
| Q5VST9 | OBSCN | ✓ | **0.038** | 0.026 | -1.45 |
| P02763 | ORM1 | ✓ | **0.012** | 0.032 | -1.42 |
| Q15293 | RCN1 | ✓ | **0.015** | 0.032 | -1.39 |
| P05109 | S100A8 | ✓ | **0.022** | 0.02 | -1.43 |
| P35542 | SAA4 | ✓ | **0.034** | 0.047 | -1.7 |
| P49908 | SELENOP | ✓ | **0.039** | 0.056 | 1.87 |
| P01011 | SERPINA3 | ✓ | **0.022** | 0.032 | -1.39 |
| P08697 | SERPINF2 | ✓ | **0.022** | 0.042 | 1.85 |
| P05155 | SERPING1 | 🗶 | 0.065 | **0.047** | -1.84 |
| Q7RTX0 | TAS1R3 | ✓ | **0.009** | 0.014 | 2.08 |
| Q15582 | TGFBI | ✓ | **0.015** | 0.022 | 1.99 |
| Q9HCJ0 | TNRC6C | ✓ | **0.031** | 0.047 | 1.9 |
| Q9H497 | TOR3A | ✓ | **0.028** | 0.032 | 1.61 |
| P19320 | VCAM1 | ✓ | **0.008** | 0.007 | -1.7 |
| P04275 | VWF | ✓ | **0.022** | 0.026 | -1.49 |
| Q6ZQQ6 | WDR87 | ✓ | **0.009** | 0.007 | -1.88 |

Values obtained from false discovery rate (FDR). Student’s and Wilcoxon tests were used for normal and non-normal variables, respectively (the corresponding value is highlighted in bold in each case). Positive Log2 fold change (FC) values indicate a higher expression in the healthy centenarians compared with the control group (green background), whereas negative values indicate a lower expression (red background). Abbreviations: A1BG, alpha-1-B glycoprotein; ABCF1, ATP binding cassette subfamily F member 1; ACOT4, acyl-coenzyme A thioesterase 4; AFM, afamin; ALB, albumin; AMPB, alpha-1-microglobulin/bikunin precursor; AOPEP, aminopeptidase; ARRB2, arrestin beta 2; AZGP1, zinc-alpha-2-glycoprotein; C1S, complement component 1S; C5, complement component C5; C7, complement component C7; C9, complement component C9; CD14, cluster of differentiation 14; CDKL1, cyclin dependent kinase like 1; CLEC3B, C-type lectin domain family 3 member B; COX18, cytochrome c oxidase assembly factor; CRISP3, cysteine-rich secretory protein 3; CRTAC1, cartilage acidic protein 1; CST3, cystatin 3 (formerly known as gamma trace, post-gamma-globulin, or neuroendocrine basic polypeptide); ECM1, extracellular matrix protein 1; EFEMP1, EGF-containing fibulin-like extracellular matrix protein 1; HPX, hemopexin precursor; IGFALS, insulin like growth factor binding protein; IGKV1D-33, immunoglobulin kappa variable 1D-33; ING2, inhibitor of growth protein 2; ITGAL, integrin alpha L chain; ITIH3, inter-alpha-trypsin inhibitor heavy chain 3; LCP1, lymphocyte cytosolic protein 1; LRG1, leucine rich alpha-2-glycoprotein 1; LYZ, lysozyme; MYH13, myosin heavy chain 13; OB2M, beta-2-microglobulin; OBSCN, obscurin; ORM1, orosomucoid 1 (also known asalpha-1-acid glycoprotein); RCN1, reticulocalbin 1; S100A8, S100 calcium binding Protein A8; SAA4, serum amyloid A4; SELENOP, selenoprotein P; SEPRINA3, serpin family A member 3; SERPING1, serpin peptidase inhibitor, clade G (C1 inhibitor), member 1 ; SERPINF2, serpin family F member 2; TAS1R3, taste 1 receptor member 3; TGFBI, transforming growth factor beta induced; TNRC6C, trinucleotide repeat containing 6C; TOR3A, torsin family 3 member A; VCAM1, vascular cell adhesion molecule 1; VWF, von Willebrand factor; WDR87, WD repeat-containing protein 87.
