# Supplementary material for: Successful aging: insights from proteome analyses of healthy centenarians
Source: Aging (Albany NY). 2020 Feb 25;12(4):3502–15. doi: 10.18632/aging.102826 (PMC7066932; doi:10.18632/aging.102826)
Supplement: Supplementary Table 2 [file aging-12-102826-s002..docx]

**Supplementary Table 2.** Immune system-related processes differentially expressed in healthy centenarians compared with the control group.

| Dataset source | Process Name | *Global FDR* | *Men FDR* | *Women FDR* | Related Process | Expression compared with controls |
| --- | --- | --- | --- | --- | --- | --- |
| GOPROCESS | Immune response | 0.094 | - | **-** | Immune response | ↑ |
| GOPROCESS | Leukocyte migration | **0.003** | - | - | Immune response | ↑ |
| GOFUNCTIONID | Immunoglobulin receptor binding | 0.069 | - | **0.007** | B-cell mediated immune response | ↑ |
| GOLOCATION | Immunoglobulin complex, circulating | - | - | 0.057 | B-cell mediated immune response | ↑ |
| BEDPATH | B cell antibody production | - | - | **0.015** | B-cell mediated immune response | ↑ |
| GOPROCESS | B cell receptor signaling pathway | **0.012** | - | **0.004** | B-cell mediated immune response | ↑ |
| GOPROCESS | Positive regulation of B cell activation | **0.012** | - | **0.009** | B-cell mediated immune response | ↑ |
| GOFUNCTIONID | Antigen binding | **<0.001** | **0.017** | **<0.001** | B-cell mediated immune response | ↑ |
| GOPROCESS | Fc-epsilon receptor signaling pathway | **<0.001** | 0.093 | **0.005** | B-cell mediated immune response | ↑ |
| GOPROCESS | Fc-gamma receptor signaling pathway involved in phagocytosis | **<0.001** | - | 0.088 | Phagocytosis | ↑ |
| GOPROCESS | Phagocytosis, recognition | **0.012** | - | **0.006** | Phagocytosis | ↑ |
| GOPROCESS | Phagocytosis, engulfment | **0.028** | - | **0.011** | Phagocytosis | ↑ |
| GOPROCESS | Neutrophil degranulation | **0.049** | - | **<0.001** | Neutrophil-mediated response | ↓ |
| GOLOCATION | Tertiary granule lumen | 0.097 | - | **0.032** | Neutrophil-mediated response | ↓ |
| GOLOCATION | Azurophil granule lumen | - | - | **0.036** | Neutrophil-mediated response | ↓ |
| GOLOCATION | Ficolin-1-rich granule lumen | - | - | **0.043** | Neutrophil-mediated response | ↓ |
| BEDPATH | MHC I antigen processing and presentation | - | - | **0.039** | T-cell mediated immune response | ↓ |
| BED | Acquired immunodeficiency síndrome | **0.018** | - | **0.006** | T-cell mediated immune response | ↓ |
| BEDMOTIVE | [Acquired immunodeficiency |  |  |  |  | ↓ |
|  | syndrome] Persistent immune activation | **0.004** | - | **0.008** | T-cell mediated immune response |  |
| GOPROCESS | Complement activation, alternative pathway | - | - | 0.066 | Complement-mediated cytolysis | ↓ |
| GOPROCESS | Cytolysis | 0.089 | - | 0.088 | Complement-mediated cytolysis | ↓ |
| GOLOCATION | Membrane attack complex | - | - | 0.055 | Complement-mediated cytolysis | ↓ |
| BED | Inflammation | - | - | **0.010** | *Inflammaging* | ↓ |
| BEDMOTIVE | [Inflammation] Chemical mediators of inflammation | - | - | **0.027** | *Inflammaging* | ↓ |
| TRRUST | Nuclear factor NF-kappa-B p105 subunit (NFKB1) | 0.054 | **-** | **-** | *Inflammaging* | ↓ |
| BED | Drug_hypersensitivity | **0.014** | - | **<0.001** | Autoimmunity | ↓ |
| BEDMOTIVE | [Drug Hypersensitivity] Immune response-drug hypersensibility | **0.010** | - | **<0.001** | Autoimmunity | ↓ |
| BED | Rheumatoid arthritis | **0.014** | - | **0.004** | Autoimmunity | ↓ |
| BEDMOTIVE | [Rheumatoid Arthritis] Articular  destruction | **0.036** | - | **0.017** | Autoimmunity | ↓ |
| BED | Nephrosis | **0.005** | - | **0.002** | Autoimmunity | ↓ |
| BEDMOTIVE | [Nephrosis] Immune complex  formation | **0.006** | - | **<0.001** | Autoimmunity | ↓ |
| BED | Systemic lupus erythematosus | **0.027** | - | 0.097 | Autoimmunity | ↓ |
|  | 05322_Systemic lupus erythematosus | 0.055 | **-** | 0.065 | Autoimmunity | ↓ |
| BED | Lupus erythematosus Discoid | **0.006** | **0.013** | 0.080 | Autoimmunity | ↓ |
| BEDMOTIVE | [Lupus_Erythematosus_Discoid]  Impaired apoptotic clearance | 0.088 | **-** | - | Autoimmunity | ↓ |
| BED | Multiple sclerosis | **0.020** | - | **0.046** | Autoimmunity | ↓ |
| BEDMOTIVE | [Multiple_sclerosis] Autoreactivity and multifocal inflammation | 0.063 | **-** | - | Autoimmunity | ↓ |
| BEDMOTIVE | [Multiple_Sclerosis] Axonal damage and neuronal loss | - | **-** | 0.061 | Autoimmunity | ↓ |
| BED | Thrombocytopenia_Immune_Primary | - | - | 0.099 | Autoimmunity | ↓ |
| GOFUNCTIONID | Protein-containing complex binding | **0.012** | 0.061 | - | Autoimmunity | ↓ |
| BED | Infection | **0.001** | **0.014** | **<0.001** | Infection | ↓ |
| GOPROCESS | Toll-like receptor signaling pathway | - | - | 0.076 | Infection | ↓ |
| BED | Septic_Shock | **0.005** | - | **<0.001** | Infection | ↓ |
| BEDMOTIVE | [Septic Shock] Systemic inflammatory response (SIRS) | **0.003** | - | **<0.001** | Infection | ↓ |
| BED | Malaria immune response | - | - | **0.006** | Infection | ↓ |
| BEDMOTIVE | [Malaria_Immune_Response] Blood-  Stage Immunity - Innate | - | - | 0.086 | Infection | ↓ |
| BED | Myocarditis | **0.006** | - | **<0.001** | Infection | ↓ |
| BEDMOTIVE | [Infection; Myocarditis] Innate immune response | **0.006** | - | **<0.001** | Infection | ↓ |
| KEGG | Staphylococcus aureus infection | **0.025** | - | **0.002** | Infection | ↓ |
| KEGG | Pertussis | **0.012** | - | **0.008** | Infection | ↓ |
| BED | Macular degeneration | **<0.001** | **0.017** | **<0.001** | Immune response-related diseases | ↓ |
| BEDMOTIVE | [Macular degeneration] Immunological  processes: chronic inflammation | **0.003** | **0.033** | **<0.001** | Immune response-related diseases | ↓ |
| BED | Diabetic retinopathy | 0.055 | **-** | **-** | Immune response-related diseases | ↓ |
| BED | Gout | - | - | 0.084 | Immune response-related diseases | ↓ |
| BEDMOTIVE | [Gout] Inflammation induced by  monosodium urate (MSU) crystals | 0.099 | - | **0.025** | Immune response-related diseases | ↓ |
| BED | Periodontitis | **0.027** | - | **0.027** | Immune response-related diseases | ↓ |
| BEDMOTIVE | [Periodontitis] Gingival inflammation | 0.094 | - | **-** | Immune response-related diseases | ↓ |
| BEDMOTIVE | [Periodontitis] Activation of osteoclasts and bone resorption | **-** | - | 0.090 | Immune response-related diseases | ↓ |
| BED | Urticaria | - | - | **0.048** | Immune response-related diseases | ↓ |
| BED | Colitis | **0.020** | - | **0.006** | Immune response-related diseases | ↓ |
| BED | Dermatitis | **-** | - | 0.067 | Immune response-related diseases | ↓ |
| BED | Obesity | **<0.001** | - | **0.013** | Immune response-related diseases | ↓ |
| BEDMOTIVE | [Obesity] Inflammation Obesity | **0.006** | - | **<0.001** | Immune response-related diseases | ↓ |
| BEDMOTIVE | [Obesity] Increase in free fatty acids | **-** | - | 0.097 | Immune response-related diseases | ↓ |

FDR q-values <0.5 are highlighted in bold, and those between 0.05 and 0.1 are in grey font. Symbols: ↑, ↓ higher and lower expression in healthy centenarians, respectively, compared with controls (also denoted by green and red background, respectively)
